# Analysis of the chemical composition and biological activity of secondary residues of Turkish Gall treated by semi-bionic technology

**DOI:** 10.1186/s40643-023-00624-9

**Published:** 2023-01-21

**Authors:** Shan Jiang, Sha Zhang, Xiangdong Jiang, Shuge Tian

**Affiliations:** 1 College of Traditional Chinese Medicine, Xinjiang Medical University, Shangde North Road, Shuimogou District, Urumqi, 830054 China; 2 The Fifth Clinical Medical College, Xinjiang Medical University, Shangde North Road, Shuimogou District, Urumqi, 830054 China

**Keywords:** Antioxidant, Antibacterial, Gallic acid, Secondary residues, Semi-bionic technique

## Abstract

In order to meet the contemporary concept of sustainable development, the reuse of biological waste has also been emphasized. Lots of papers nowadays study the extraction of primary residues. The disposal of secondary residues is often neglected. The chemical composition and biological activity of secondary residues of Turkish Gall (SRTG) were researched in this paper. We selected five methods to extract the SRTG, and the extraction conditions were water, hydrochloric acid buffer (pH = 2), artificial gastric juice (pH = 2), phosphate buffer (pH = 6.8), and artificial intestinal solution (pH = 6.8). The changes of phenolic components were determined by spectrophotometry and high-performance liquid chromatography. The acid-base environment did not affect total polyphenols contents and gallic acid ethyl ester contents in SRTG. But it affected the gallic acid contents in SRTG. The contents of gallic acid in the hydrochloric acid buffer extraction groups were 1.63 times that of the water extraction group. The SRTG were extracted by hydrochloric acid buffer also had better inhibition on *Escherichia coli* and *Staphylococcus aureus*. In addition, SRTG showed positive effects on 1,1-Diphenyl-2-picrylhydrazyl Free, 2,2′-azino-bis (3-ethylbenzothiazoline-6-sulfonic acid), ·OH radicals, and Ferric ion reducing antioxidant power. Some active components of SRTG can be effectively released through the digestion of simulated gastric juices in vitro. The change of active ingredients affects the antibacterial and antioxidant capacity. The results provide data support for the conversion of secondary residues into products, such as feed additives. The SRTG has certain contributes to the value of the circular economy.

**Graphical Abstract:**

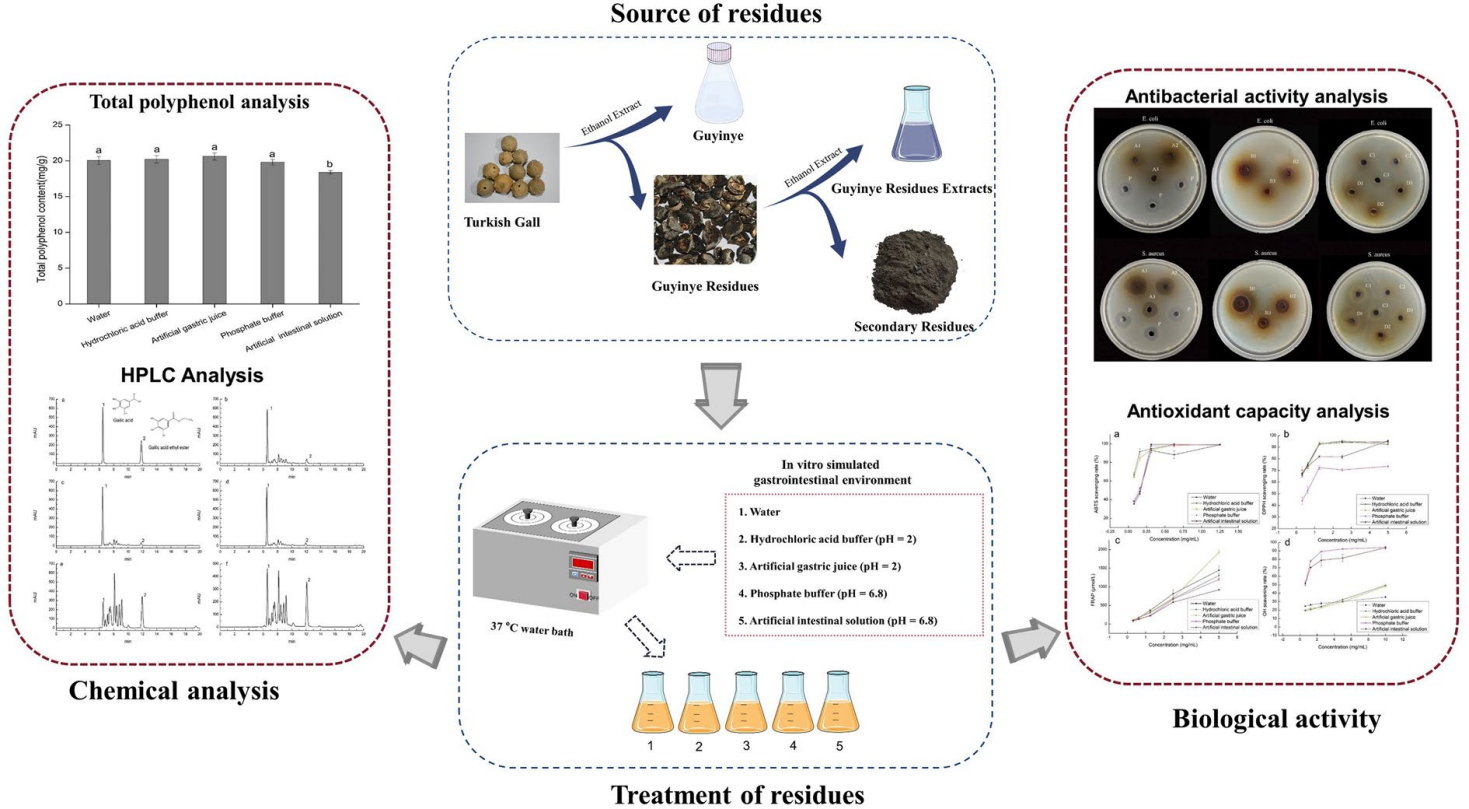

## Introduction

The residues generated during the extraction and processing of natural medicines have become a major issue threatening ecosystems and long-term development. Approximately 70% of the residues are produced by the production process of Chinese patent medicines. The general methods of disposing residues are burial and incineration. Serious problems, such as environmental pollution, are caused by the unreasonable handling of residues. The study found that most Chinese herbal residues are rich in organic compounds and trace elements (Lu and Li 2021). At present, Chinese herbal residues are gradually becoming a hot topic of research. Chinese herbal residues are applied in various fields, such as industry, agriculture, and animal husbandry (Bi et al. 2022; Zhao and Zhou 2016).

Chemical components are the material foundation for studying disease prevention and therapy in Chinese medicine. The semi-bionic extraction procedure was developed in order to maximize the medicine's efficacy. It is based on a gray thinking model with the biological activity of the extract as a guide. The method simulates the environment of medicine transport and absorption through the gastrointestinal tract. Active ingredients in Chinese herbal medicines are extracted with different pH solvents and enzymes. This method avoids the destruction of the active ingredients by high temperatures. It also facilitates the dissolution and bio-transformation of the active ingredients (Zhou et al. 2023). The semi-bionic extraction method has been chosen to study the physiological effects of complex digestive processes of food or drug (Yan et al. 2022). This has led to the rapid development of research and wider application of semi-bionic technology in the direction of Chinese medicine extraction and bio-availability of nutrients. Meanwhile, many studies have shown that semi-bionic extraction can get more active ingredients than traditional extraction methods (Guo and Zhou 2015). It also shows that this method has good prospects for application in the field of Chinese medicine extraction.

Turkish Gall is *Cynips gallae-tinctoriae Olivier* larval parasite produced galls on young branches of *Quercus infectoria Olivier* (Fagaceae). Turkish Gall is a typical medicine that has a large number of phenolic components. The studies indicate gallic acid and its derivatives have pharmacological effects, such as antioxidant and antibacterial effects (Al Zahrani et al. 2020; Hossain et al. 2020). Gallic acid can improve intestinal health and is used as feed additives (Moussa et al. 2021; Salaheen et al. 2017). Guyinye is a kind of pharmaceutical product, which is made from Turkish Gall. It has antibacterial, anti-inflammatory, and analgesic effects (Qi et al. 2022). The research finds that the Guyinye residues still contain a large amount of active ingredients and have value for utilization (Jiang et al. 2021). The Guyinye residues extracts obtained from the optimized extraction method are still contain gallic acid and ellagic acid (Zeng et al. 2019). Biochar made from Turkish Gall residues is discovered to adsorb phenol pollutants and realize the resource utilization of waste residues (Xie et al. 2020).

Although trials showed that the active ingredient was still contained in the primary residues of Turkish Gall, no validation carried out on its secondary residues. In this paper, SRTG were studied by semi-bionic technique, to understand the active component changes of SRTG after simulated digestion. We also studied its effect on antibacterial effect and antioxidant capacity. This will provide data to the subsequent development of secondary residues into other products.

## Materials and methods

### Materials and chemicals

2,4,6-Tri(2-pyridyl)-1,3,5-triazine (TPTZ), 1,1-Diphenyl-2-picrylhydrazyl Free (DPPH), 2,2′-azino-bis (3-ethylbenzothiazoline-6-sulfonic acid) (ABTS), Nutrient Broth, Agar Powder, and Trypsin (1:250) are from Beijing Solarbio Science & Technology Co., Ltd. Pepsin (1:3000) from BioFroxx, Germany. *Escherichia coli* (*E. coli*) is used as standard quality control strain ATCC 25922 and *Staphylococcus aureus* (*S. aureus*) is used as standard quality control strain ATCC 25923. Penicillin sodium is from Shanghai yuanye Bio-Technology Co., Ltd, Lot: S15J7Y9073, mass fraction 97%. Gallic acid is from Chengdu Kelong Chemical Reagent Factory, mass fraction 99%. Gallic acid ethyl ester is from Chengdu Prefa Technology Development Co. Lot: PRF20090442, mass fraction 98%. Folin–Ciocalteu’s phenol reagent is a homemade laboratory product. Other chemical reagents are all of analytical purity grade. Methanol is chromatographically pure grade. Water is purified water.

### Raw material

**Fig.1:**
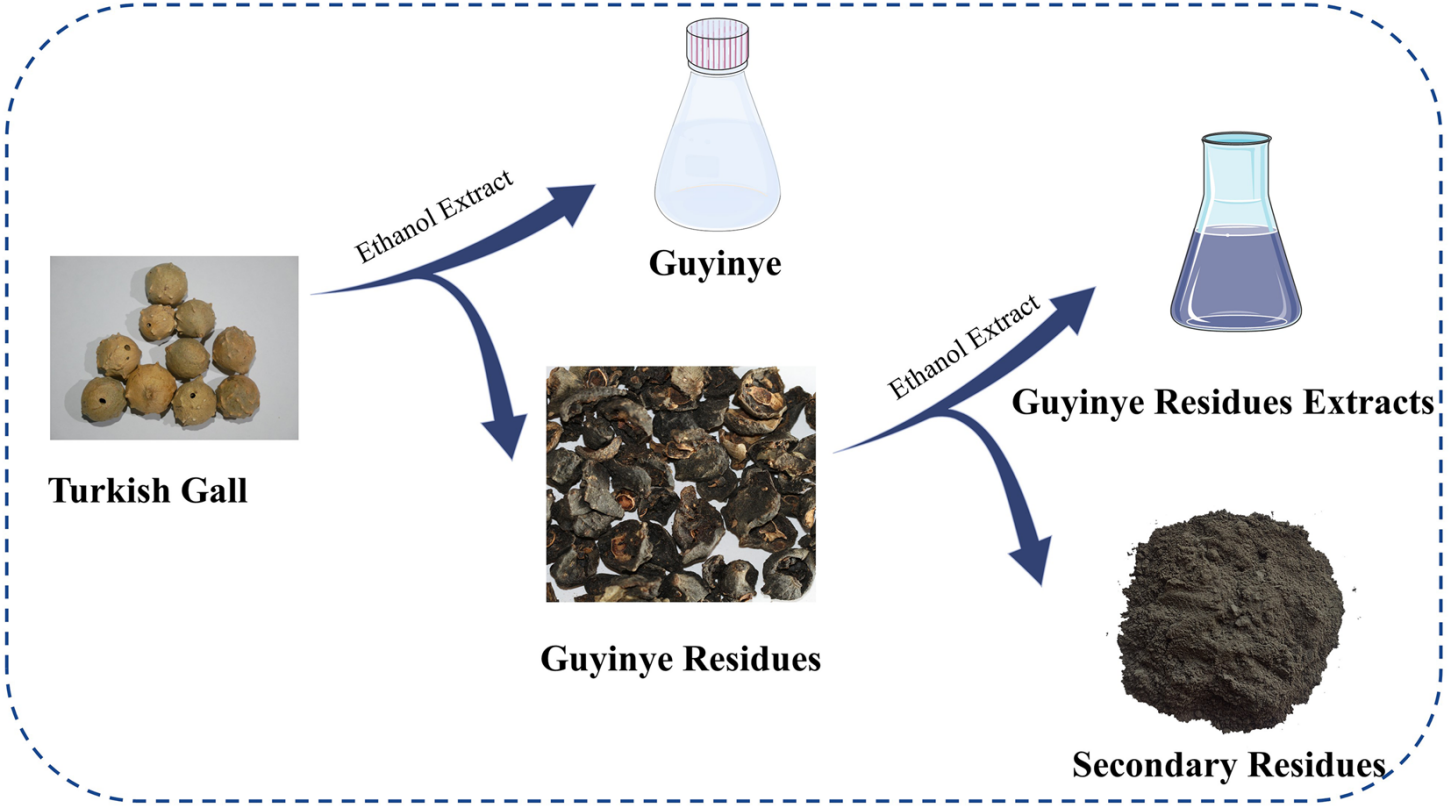
Source of raw material

The Guyinye was prepared by extracting Turkish Gall with a certain concentration of ethanol. The Guyinye residues from Xinjiang Qimu Medical Research Institute were extracted with 60% ethanol to obtain secondary residues (Zeng et al. 2019). Secondary residues were dried, crushed, and sieved through 200 mesh to obtain the raw material (Fig. 1).

### Preparation of working solutions

Dilute hydrochloric acid: Concentrated hydrochloric acid (234 mL) was diluted to 1000 mL with water.

Potassium dihydrogen phosphate buffer: Potassium dihydrogen phosphate (6.8 g) was added with water to obtain 500 mL buffer solution. The pH of the buffer solution was adjusted to 6.8 by using 0.1 mol/L sodium hydroxide.

Artificial gastric juice (pH = 2): The dilute hydrochloric acid (16.4 mL), 800 mL water, and 10 g pepsin were added to volumetric flask (1000 mL). The mixtures were shaken well and diluted to 1000 mL with water (Zhu et al. 2019).

Artificial intestinal solution (pH = 6.8): Potassium dihydrogen phosphate buffer (500 mL) and trypsin (10 g) were added to volumetric flasks (1000 mL). The solutions were mixed and diluted to 1000 mL with water (Chen et al. 2018).

Hydrochloric acid buffer (pH = 2) was prepared in the same way as artificial gastric juice without the addition of digestive enzymes.

Phosphate buffer (pH = 6.8) was prepared in the same way as artificial intestinal solution without the addition of digestive enzymes.

### Treatment procedure

SRTG (1 g) was added with 50 mL of working solutions (Lin et al. 2021). The mixtures were kept in a constant-temperature and lightproof water bath at 37 °C for 30 min and filtered to obtain extracts. The extracts were diluted to determine the total polyphenols content (TPC). The extracts were volatilized, dissolved with sterile water, passed through 0.22 μm sterile filter membrane, and used for bacteriostatic experiments. The extracts were extracted with ethyl acetate three times (25 mL), and then upper clear layer obtained from the three extractions was combined, evaporated, dissolved in methanol, passed through 0.22 μm membrane, and used for high-performance liquid chromatography (HPLC) experiments.

### Determination of the TPC

We referred to the Pharmacopoeia of the People’s Republic of China (The Commission of Pharmacopoeia 2020). Sample solutions (2 mL), Folin-Ciocalteu’s phenol reagent (1 mL), and 15% Na_2_CO_3_ (13 mL) were added to a 25 mL volumetric flask, and diluted to the mark with water, shaken well, and placed for 30 min. The absorbance was measured at 760 nm. Blank controls were prepared using the method above.

### HPLC analysis

Standard solutions of gallic acid and gallic acid ethyl ester were prepared, passed through 0.22 μm filter membrane, and stored at 4 °C. Separation was performed using HPLC with a diode array detector (HPLC-DAD) apparatus equipped with the WondaSil C18 column (4.6 × 250 mm, 5 μm) and set column temperature as 30 °C. The mobile phase was modified referred previous method (Majumdar et al. 2021). The solvent was consisted of solutions A (methanol) and B (0.2% phosphoric acid water). The gradient program was as follows: 0–3 min, 80% B–57% B; 3–7 min, 57% B–55% B; 7–11 min, 55% B–54% B; 11–14 min, 54% B–51% B; 14–15 min, 51% B–45% B; and 15–20 min, 45% B–35% B. Analyses were performed at a flow rate of 0.8 mL/min, and the detected wavelength was 273 nm with an injection volume of 2 μL.

### Inhibitory activity of SRTG against Escherichia coli and Staphylococcus aureus

*E. coli* and *S. aureus* are the main pathogenic bacteria that cause gastrointestinal diseases. The inhibition effects of the extracts were observed using the perforation method (Soliman and El-Sayed 2021). A single colony was collected from a blood plate culturing *E. coli* and *S. aureus*, placed in 2 mL nutrient broth culture, and incubated at 5% CO_2_ and 37 °C for 24 h. Suspensions of *E. coli* and *S. aureus* were diluted for counting. The bacterial solution was adjusted to 10^8^ CFU/mL. Suspensions of *E. coli* and *S. aureus* (100 μL, 10^8^ CFU/mL) were collected and spread onto agar plates with a glass triangular stick. 6 mm holes were punched on the petri dish by using a hole puncher (Javed et al. 2020). Sample solutions (40 µL) were injected into the well. Penicillin sodium solution was positive control solution and solvent was blank control solution. Petri dish was placed in incubator 24 h to measure the diameter of the inhibition zone.

### Assessment of the minimum bactericidal concentration (MBC)

The double dilution method and streak plate method were used to determine the minimum bactericidal concentration (MBC) against *E. coli* and *S. aureus* (Wang et al. 2022). The suspensions of *E. coli* and *S. aureus* were diluted to obtain a concentration of 10^6^ CFU/mL and used in test. The lowest drug concentration for sterile growth was called the MBC. Each experiment was performed parallel in three times.

### Antioxidant capacity

The experiments were carried out with some modifications according to the previous methods (Wu et al. 2021). The antioxidant capacity of the samples was determined using the microplate reader. ABTS working solution preparation: 10 mL of 7 mmol/L ABTS solution and 5 mL of 2.45 mmol/L K_2_S_2_O_8_ were mixed to obtain ABTS stock solution. Then ABTS stock solution was kept out light for 14 h. The ABTS working solution was obtained by diluting the stock solution with ethanol to have an absorbance of 0.70 ± 0.02. The samples were diluted with water to 1250, 625, 312.5, 156.25, and 78.125 μg/mL, respectively. The above dilutions (100 μL) and ABTS working solution (100 μL) were pipetted into a 96-well plate, mixed well, and kept away from light for 10 min and the absorbance was measured at 734 nm. The scavenging rate was calculated in the following equation (Eq. (1)). A_0_ was the absorbance value of the control group (no samples), A_1_ was the test group (samples and reagent), and A_2_ was the absorbance value of the sample group (no reagent).

1 $$\mathrm{Scavenging rate }\left(\mathrm{\%}\right)=\frac{{A}_{0}-\left({A}_{1}-{A}_{2}\right)}{{A}_{0}}.$$

The DPPH scavenging assay followed the relevant literature with some modifications (Takatsuka et al. 2022; Chen et al. 2022). Samples of 5000, 2500, 1250, 625, and 312.5 μg/mL were prepared. The extracts solutions (100 μL) and 0.1 mmol/L DPPH-ethanol solution (100 μL) were taken into a 96-well plate, respectively, shaken well, and kept out light for 30 min. The absorbance of the sample solution was measured at 517 nm. The scavenging rate was calculated in the same way as Eq. (1).

Ferric ion reducing antioxidant power (FRAP) assay referenced to relevant literature with some modifications (Mocan et al. 2020). Preparation of FRAP working solution is as follows: FRAP1: Take 0.18 g of sodium acetate and 1.6 mL of acetic acid to 100 mL volumetric flask and fix to volume. FRAP2: Take 78 mg of TPTZ and fix it to 25 mL with 40 mmol/L hydrochloric acid. FRAP3: Fix 270.3 mg of 6H_2_O·FeCl_3_ in water to 50 mL volumetric flask. FRAP1: FRAP2: FRAP3 = 10:1:1, which were mixed to obtain the FRAP working solution. Pipette 10 μL of extracts solution and 200 μL of FRAP working solution into a 96-well plate, shake well, and keep away from light for 30 min. Absorbance values were measured at 593 nm. FeSO_4_ was used as a standard substance and its antioxidant capacity was expressed in terms of FeSO_4_ concentration (μmol/L).

OH radical scavenging experiment was based on the literature with some modifications (Li et al. 2022a). The extracts solutions (70 μL), 6 mmol/L FeSO_4_ solution (60 μL), and 6 mmol/L salicylic acid-ethanol solution (60 μL) were added in a 96-well plate sequentially, mixed, and placed for 10 min, and 60 μL of 6 mmol/L H_2_O_2_ solution was added, mixed, and kept out light for 30 min. Absorbance values were measured at 510 nm. The scavenging rate was calculated in the same way as Eq. (1).

### Statistical Analysis

Each group of experiment was repeated three times, and the values are expressed as mean ± standard deviation. The OriginPro version 8.5.1 was used for image reporting, and the SPSS version 21 was used for statistical data analysis. The one-way analysis of variance was used to analyze differences between samples. For the multiple comparison analysis, the Tukey test for significant differences was used. Pearson correlation analysis was also used. *p* < 0.05 indicated a significant difference.

## Results and discussion

### Total phenolic in SRTG

**Fig. 2:**
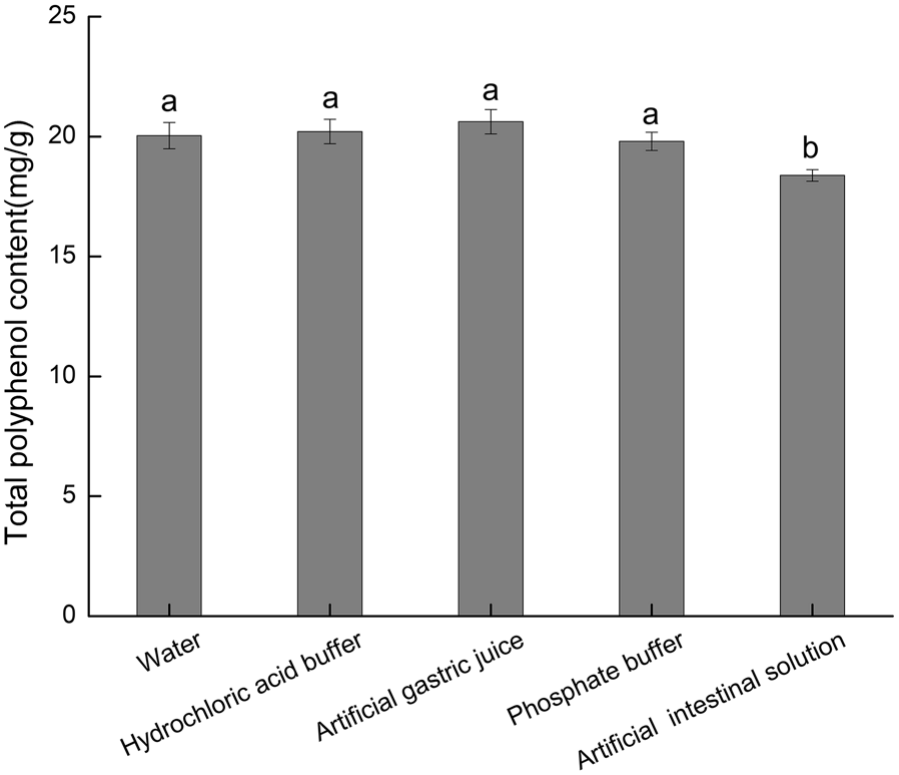
Total polyphenols content of secondary residues of Turkish Gall by different treatment conditions. Different letters (**a**, **b**) indicate significant difference (*p* < 0.05)

The polyphenol content of the plants may increase or decrease by simulated in vitro digestion, which is related to the nature of the plant ingredients (Lin et al. 2021; Zhu et al. 2020a). This paper studied the change of polyphenols contents in SRTG by different treatment conditions. Gallic acid was used as the standard. The regression equations were linear, ranging in the concentration of 1.0008–10.0080 μg/mL (*y* = 0.1115*x* + 0.0281, *r*^*2*^ = 0.9992). The TPC of SRTG obtained by different treatment conditions (water, hydrochloric acid buffer, artificial gastric juice, phosphate buffer, artificial intestinal solution) was 20.04, 20.21, 20.62, 19.80, and 18.38 mg/g, respectively. The contents of polyphenols in SRTG treated with water, hydrochloric acid buffer, artificial gastric juice, and phosphate buffer were not significant, respectively. The TPC of the artificial intestinal solution treated with SRTG was slightly lower than the other four groups. The changing of the pH value had no significant effect to the TPC. The change of TPC may be related to the type of enzyme. The TPC in SRTG by the different treatment conditions is shown in Fig. 2.

### HPLC analysis of the composition of SRTG

**Fig. 3:**
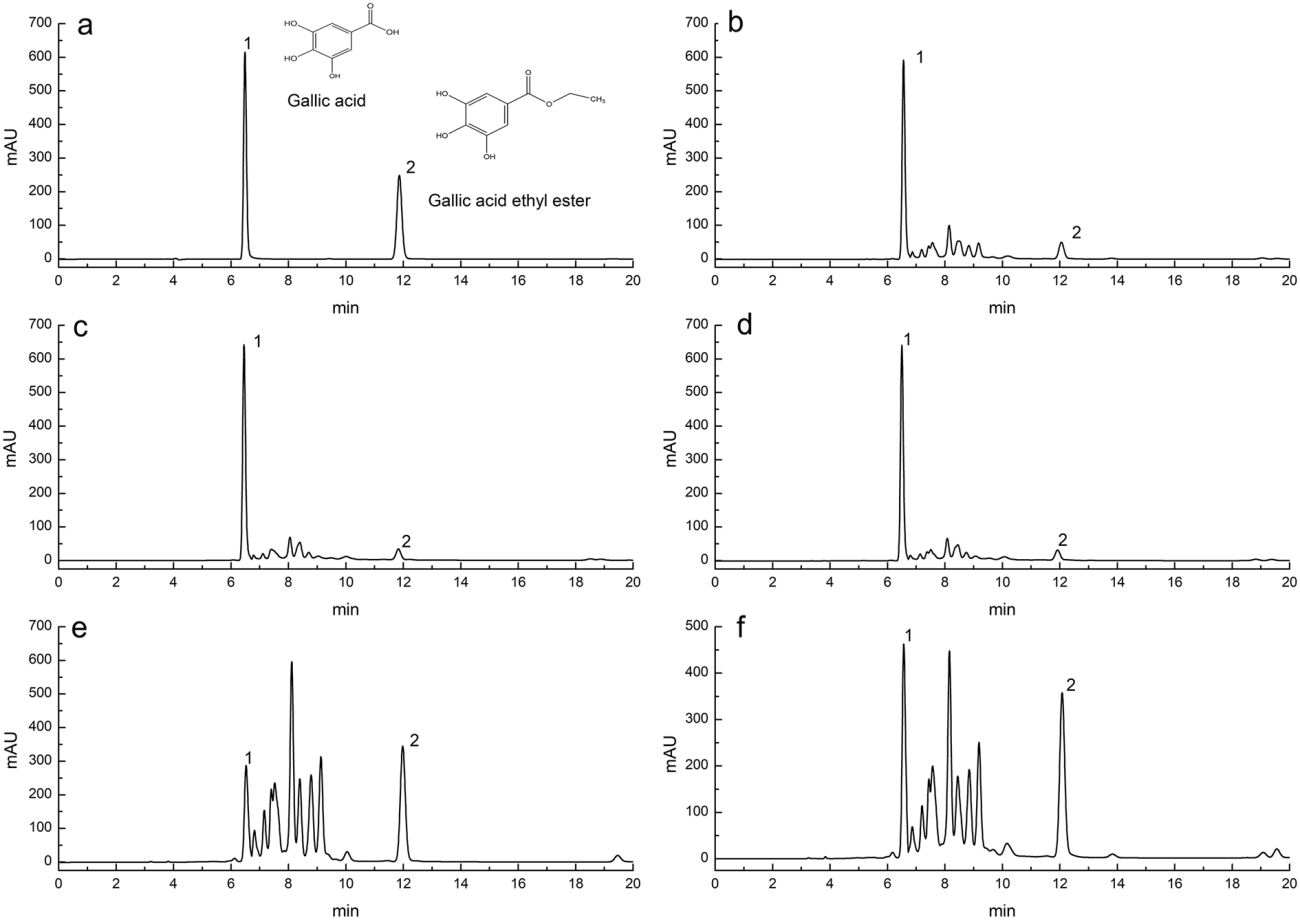
Chromatograms of secondary residues of Turkish Gall under different treatment conditions (**a** Standard solution; **b** Water treat; **c** Hydrochloric acid buffer treat; **d** Artificial gastric juice treat; **e**, Phosphate buffer treat; **f** Artificial intestinal solution treat; 1 represents gallic acid and 2 represents gallic acid ethyl ester)

Gallic acid and gallic acid ethyl ester are chemical compounds that inhibit the growth and reproduction of *E. coli* and *S. aureus* (Zhang et al. 2022). Therefore, this paper chooses these two components for determination. The results showed that the main factor affecting the releasing of gallic acid was the acidity and alkalinity of gastrointestinal environment. The SRTG were treated with hydrochloric acid buffer and artificial gastric juice, and their gallic acid contents all reached 5.30 mg/g. The SRTG were treated with water and its gallic acid contents reached 3.26 mg/g. The SRTG were treated with phosphate buffer and artificial intestinal solution, which contained gallic acid of 0.28 mg/g and 0.41 mg/g, respectively. The contents of gallic acid in the artificial gastric juice treatment groups were 1.63 times that of the water treatment group. The content of gallic acid increased significantly after SRTG was digested by simulated gastric juice in vitro. The reason for the increase in gallic acid contents may be that the gastric juice made the polyphenol macromolecules in the residues was broken and turned into small molecular substances. The solvent of phosphate buffer and artificial intestinal solution inhibited the dissolution of gallic acid, resulting in decrease in the contents of gallic acid. The acidity or alkalinity of the solvent did not significantly affect the gallic acid ethyl ester contents of SRTG. The gallic acid ethyl ester contents of SRTG varied in the range of 0.50–0.58 mg/g. We also examined the methodology of the experiments. According to International Conference on Harmonization guidelines (2022), the HPLC-DAD method was validated. The linear relationship of gallic acid and ethyl gallate is shown in Table 1. The contents of gallic acid and gallic acid ethyl ester are shown in Table 2. The chromatographic condition was chosen to provide effective separations of gallic acid and gallic acid ethyl ester in SRTG. Chromatograms are shown in Fig. 3.

**Table 1:** Linear analysis parameters of gallic acid and gallic acid ethyl ester

Standard	Linear equations	Quality range (μg)	*r*^*2*^
Gallic acid	*y* = 1170.4*x* + 24.257	0.7615–9.8995	1.0000
Gallic acid ethyl ester	*y* = 1069.9*x* + 7.3536	0.5750–7.4750	1.0000

**Table 2:** Contents of gallic acid and gallic acid ethyl ester

Working Solution	Gallic acid (mg/g)	RSD (%)	Gallic acid ethyl ester (mg/g)	RSD (%)
Water	3.26 ± 0.06	1.80	0.56 ± 0.01	1.64
Hydrochloric acid buffer	5.30 ± 0.11	2.08	0.58 ± 0.01	2.15
Artificial gastric juice	5.30 ± 0.02	0.33	0.56 ± 0.00	0.09
Phosphate buffer	0.28 ± 0.00	0.11	0.50 ± 0.00	0.21
Artificial intestinal solution	0.41 ± 0.00	1.13	0.52 ± 0.00	1.00

Data are expressed as mean ± standard deviation (*n* = 3)

### Analysis of bacteriostatic activity

**Fig. 4:**
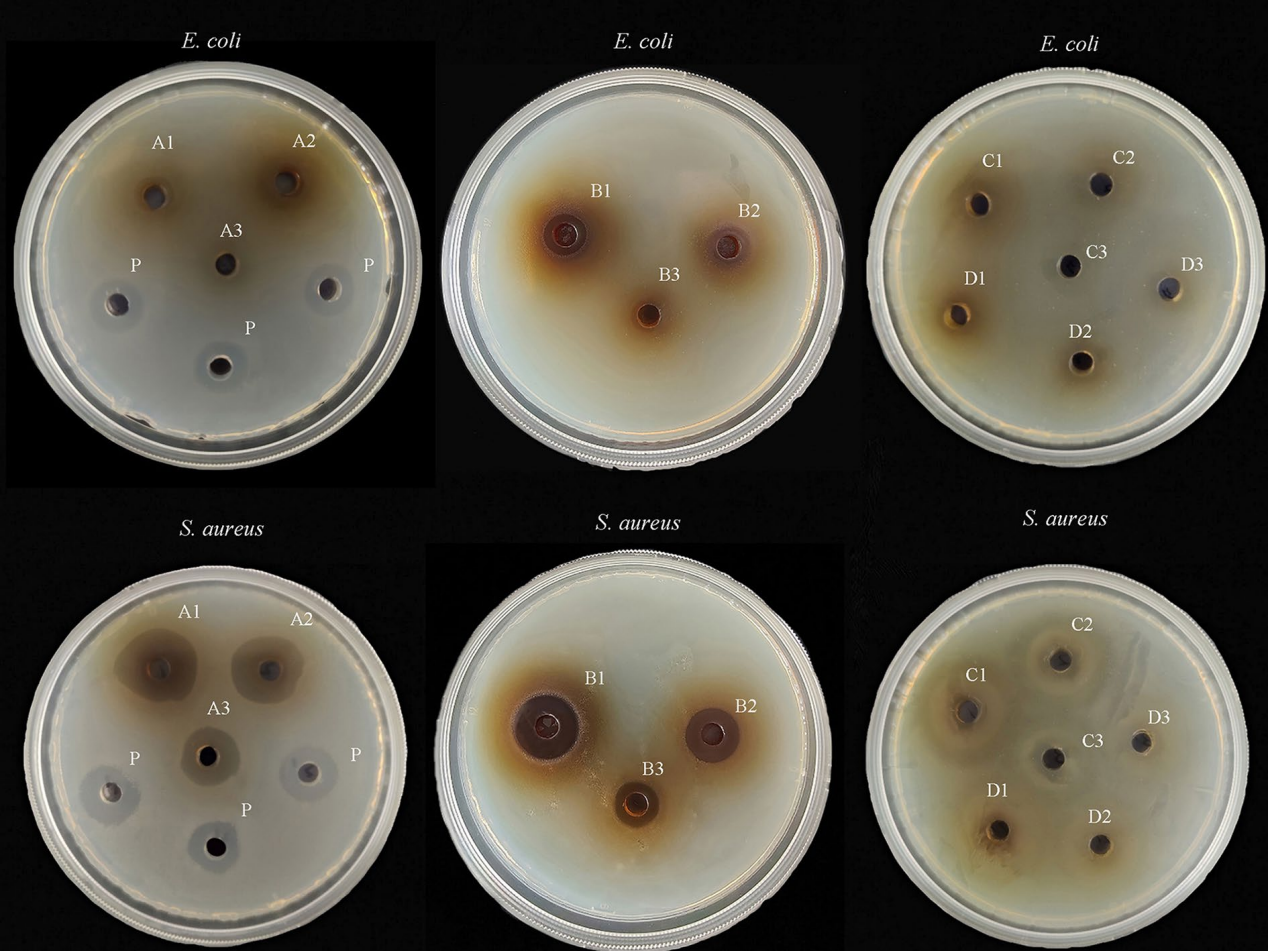
Bacterial inhibition of *E. coli* and *S. aureus* (P, Positive control; **A** Water treat; **B** Hydrochloric acid buffer treat; **C** Phosphate buffer treat; **D** Artificial intestinal solution treat. 1, 2 g/mL; 2, 1 g/mL; 3, 0.5 g/mL)

*E.coli* and *S. aureus* are common gastrointestinal pathogens. Studies have shown that Turkish Gall has the ability to inhibit *E. coli* and *S. aureus* (Zheng et al. 2017). Gallic acid, the main component in Turkish Gall, can be used as an antibacterial agent (Sheoran et al. 2019; Singh et al. 2019). No significant difference was found between the gallic acid contents of secondary residues obtained by hydrochloric acid buffer and artificial gastric juice. The artificial gastric juice as blank solution affected the growth of *E. coli* and *S. aureus*. Therefore, we selected the following four groups for the bacteriostatic experiment, water, hydrochloric acid buffer, phosphate buffer, and artificial intestinal solution, respectively. In this study, 2, 1, and 0.5 g/mL samples were prepared to observe the inhibition against *E. coli* and *S. aureus*. The SRTG were extracted by phosphate buffer and artificial intestinal solution had no inhibiting effect on the growth of *E. coli* and *S. aureus*. SRTG (0.5 g/mL) were extracted by hydrochloric acid buffer and water can inhibit the growth of *S. aureus*. The SRTG (1 g/mL) were extracted by hydrochloric acid buffer had a certain inhibition effect on the growth of *E. coli*, but the SRTG (1 g/mL) were extracted by water had no inhibiting effect on the growth of *E. coli*. The SRTG were extracted by hydrochloric acid buffer had better inhibition effect on the growth of *E. coli* than water. *In vitro* gastric and intestinal simulated digestion may alter the release of phenolic acids from SRTG. The change of phenolic acids contents in the SRTG may alter the bacteriostasis capacity. SRTG were more effective against *S. aureus* than *E. coli* (Fig. 4). It was consistent with the conclusion of the reference (Shao et al. 2015). The study indicated that in vitro simulated gastric digestion was beneficial in inhibiting the growth of *S. aureus* and *E. coli*. It provided a basis for the development of the SRTG into feed and other products to reduce the risk of gastrointestinal diseases in animals. The sizes of the inhibition zone are shown in Table 3.

**Table 3:** Inhibition zone diameters of *E. coli* and *S. aureus*

Bacteria	Working Solution	Secondary Residues of Turkish Gall			Penicillin sodium 0.25 mg/mL
		2 g/mL	1 g/mL	0.5 g/mL	
*E. coli*	Water	8.13 ± 0.32^ g^	–	–	11.37 ± 0.40
	Hydrochloric acid buffer	11.20 ± 1.41^ef^	8.03 ± 1.31^ g^	–	
	Phosphate buffer	–	–	–	
	Artificial intestinal solution	–	–	–	
*S. aureus*	Water	19.40 ± 1.06^a^	16.53 ± 0.65^b^	14.07 ± 0.70^ cd^	14.77 ± 0.50
	Hydrochloric acid buffer	15.63 ± 0.59^bc^	12.33 ± 0.31^de^	9.93 ± 0.15^ fg^	
	Phosphate buffer	–	–	–	
	Artificial intestinal solution	–	–	–	

The diameter of the inhibition zone includes the diameter of the punched hole (6.0 mm). Different letters (a, b, c, d, e, f, g) indicate significant differences (*p* < 0.05, Tukey test). – no inhibition zone formed

### Assessment of MBC

The susceptibility testing showed that hydrochloric acid buffer extracts had significant effect on *E. coli* and *S. aureus*. The hydrochloric acid buffer extracts were used to determine the MBC of *E. coli* and *S. aureus*. No bacterial growth was observed at medicine concentration of 0.125 g/mL. Bacterial growth was observed at medicine concentration of 0.0625 g/mL. Therefore, the MBC for *E. coli* was 0.125 g/mL. The MBC for *S. aureus* was 0.125 g/mL.

### Analysis of antioxidant capacity

Antioxidants can reduce the radical oxidation reaction on the body cell and tissue damage, delay cell senescence, and other effects (Li et al. 2022b). In order to verify comprehensively the effect of these five treatment methods on antioxidant activity, four antioxidant methods were selected for evaluation.

**Fig. 5:**
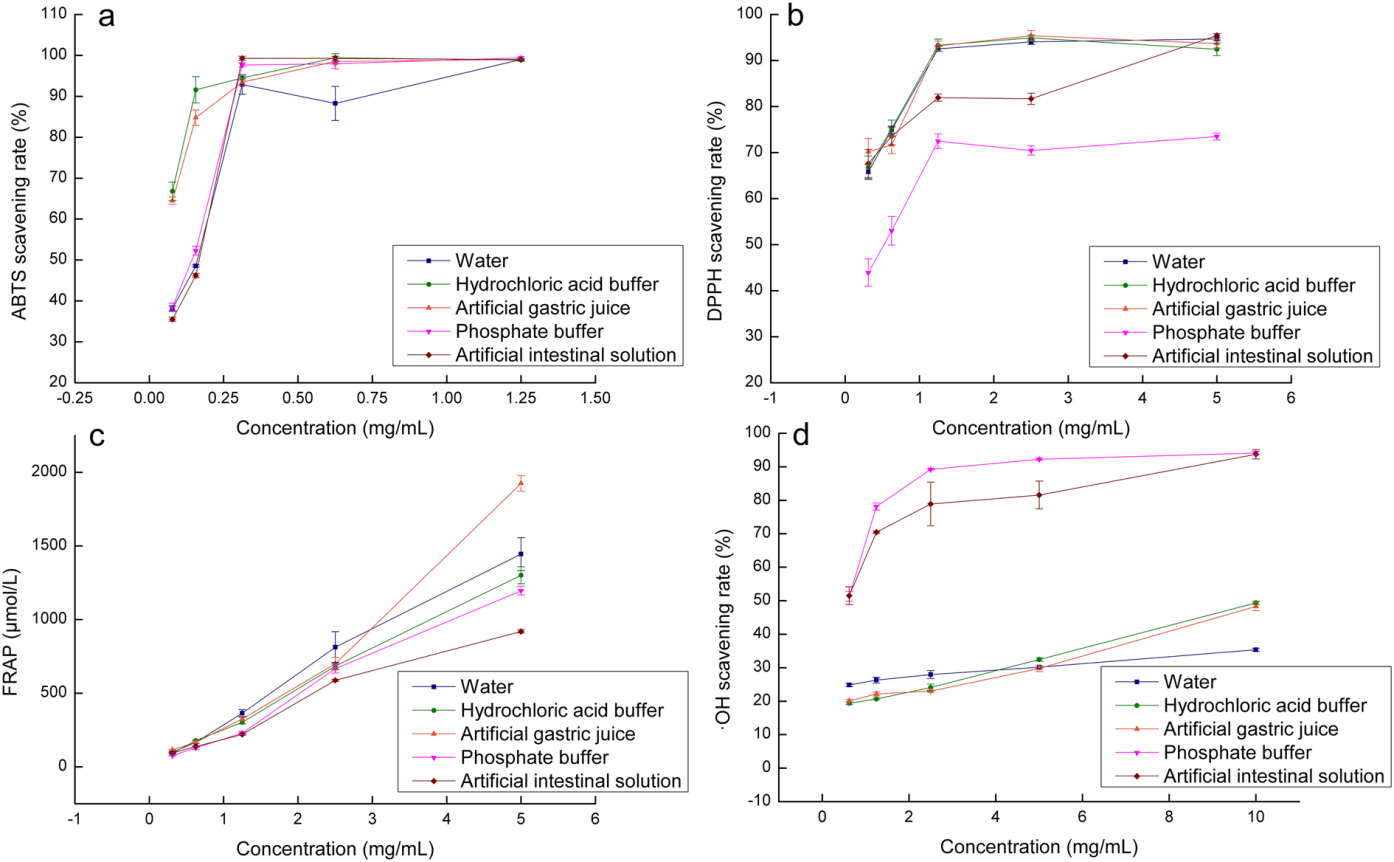
Antioxidant capacity of the secondary residues of Turkish Gall by five treatment methods. (**a** ABTS radicals scavenging capability; **b** DPPH radicals scavenging capability; **c** Ferric ion reducing antioxidant power; **d** OH radical scavenging capacity)

The results for ABTS scavenging ability are shown in Fig. 5a. SRTG (0.3125 mg/mL) were the inflection point. When the concentrations of SRTG were greater than 0.3125 mg/mL, the ABTS scavenging ability of all treatment groups was more than 90%. When the concentrations of SRTG were less than 0.3125 mg/mL, the antioxidant capacity showed a decreasing trend as the concentration of residues decreased. The hydrochloric acid buffer and artificial gastric juice treatment groups (concentrations  < 0.3125 mg/mL) had higher ability to scavenge ABTS radical than the water, phosphate buffer, and artificial solution treatment groups. The study found that *in vitro* gastric simulated digestion promoted the release of phenolic acids from SRTG. The change of phenolic acids contents in the SRTG altered the ABTS radical scavenging ability.

Figure 5b shows the results of the DPPH radical scavenging ability. The phosphate buffer group had less ability to scavenge DPPH radicals than the other four groups. The scavenging rate achieved 73.49% by the phosphate buffer treatment group at the concentration of 5 mg/mL. The DPPH scavenging rate could achieve above 90% in the other four groups. Although the five treatment methods all had good effects on DPPH scavenging, different treatments had different scavenging abilities. The DPPH radical scavenging ability was the result of the synergistic and antagonistic effects of all antioxidant substances. The changes of gastrointestinal environment may affect the structure and interaction of antioxidant substances.

There was a dose–effect relationship between FRAP and concentration. SRTG had a fine ability for FRAP (Fig. 5c). The concentration of FeSO_4_ (μmol/L) was used as the horizontal coordinate and the absorbance as the vertical coordinate. The standard curve was *y* = 0.0007*x* + 0.1296, *r*^*2*^ = 0.9998. The FRAP was diminished as the sample concentration decreased. SRTG by water, hydrochloric acid buffer, and artificial gastric juice treat had slightly higher reducing power than phosphate buffer and artificial intestinal solution.

The results for ·OH radical scavenging capacity are shown in Fig. 5d. The phosphate buffer and artificial intestinal solution treatment groups. The scavenging rate reached 90% in the phosphate buffer and artificial intestinal solution treatment groups at the concentration of 10 mg/mL. The antioxidant capacity reached 35.39%, 49.39%, and 48.31% in water, hydrochloric acid buffer, and artificial gastric juice treatment groups (10 mg/mL), respectively. The reason that intestinal digestion had better ·OH radical scavenging capacity than gastric juice digestion may be related to the acid-base environment and types of enzymes.

The SRTG treated by five methods had outstanding antioxidant capacity. After simulated digestion of stomach and intestine, the existence form and release amount of phenolic acid might be changed. So antioxidant activities were altered. However, the reason for the antioxidation mechanism of SRTG by simulating gastrointestinal digestion is not explicit; it needs to be studied further.

### Correlation analysis between the TPC and antioxidant activity

Table 4 shows the correlations between DPPH, ABTS, OH radicals, FRAP, and TPC. The absolute value is closed to 1, indicating a stronger correlation. The absolute value is closed to 0, indicating a less correlation (Zhu et al. 2020b). TPC did not necessarily correlate with antioxidant activity (Peng et al. 2016). In this study, the correlation coefficients between TPC and antioxidant activity were ·OH (− 0.718), FRAP (0.707), DPPH (0.510), and ABTS (− 0.086). The results showed that TPC was correlated with ·OH and FRAP. However, TPC has little correlation with DPPH and ABTS. There are many factors determining antioxidant activity. It depends not only on the bioactive components but also on the source of phenols. It needs to be considered comprehensively. Moreover, the antioxidant mechanism of Chinese medicine is more complex and needs to be further studied.

**Table 4:** Correlation analysis between TPC and antioxidant activity

Indicators	TPC	ABTS	DPPH	FRAP	OH
TPC	1.000	− 0.086	0.510	0.707	− 0.718
ABTS		1.000	− 0.874	− 0.508	0.739
DPPH			1.000	0.841	− 0.962^**^
FRAP				1.000	− 0.895^*^
OH					1.000

^*^indicates significant, *p* < 0.05; ^**^indicates highly significant, *p* < 0.01

TPC Total polyphenols content, ABTS, 2, 2′-azino-bis (3-ethylbenzothiazoline-6-sulfonic acid), DPPH 1,1-Diphenyl-2-picrylhydrazyl Free, FRAP Ferric ion reducing antioxidant power

## Conclusions

In this paper, five methods were used to extract SRTG. The research found that SRTG still contained active ingredients, such as TPC, gallic acid, and gallic acid ethyl ester. The contents of gallic acid in SRTG treated with the hydrochloric acid buffer and artificial gastric juice were the highest. The contents could all reach 5 mg/g. The hydrochloric acid buffer and artificial gastric juice can promote the release of gallic acid in SRTG compared with other extraction conditions. Meanwhile, the SRTG extracted with hydrochloric acid buffer can inhibit effectively the growth of *E. coli* and *S. aureus*. The SRTG were more effective against *S. aureus* than *E. coli* under the same extraction conditions. In addition, the SRTG had better scavenging effect on variety of radicals by simulating gastric and intestinal digestion in vitro. The results show that SRTG still have large potential to be utilized in production of biotechnological products with added value, which has certain contributes to the value of the circular economy.

## Data Availability

The datasets generated during and/or analyzed during the current study are available from the corresponding author on reasonable request.

## Abbreviations

SRTG: Secondary residues of Turkish Gall
HPLC: High-performance liquid chromatography
TPC: Total polyphenols content
DAD: Diode array detector
MBC: Minimum bactericidal concentration
DPPH: 1,1-Diphenyl-2-picrylhydrazyl Free
ABTS: 2,2′-Azino-bis (3-ethylbenzothiazoline-6-sulfonic acid)
FRAP: Ferric ion reducing antioxidant power
TPTZ: 2,4,6-Tri(2-pyridyl)-1,3,5-triazine
